# Conducting Polymer Nanostructures: Template Synthesis and Applications in Energy Storage

**DOI:** 10.3390/ijms11072636

**Published:** 2010-07-02

**Authors:** Lijia Pan, Hao Qiu, Chunmeng Dou, Yun Li, Lin Pu, Jianbin Xu, Yi Shi

**Affiliations:** 1 National Laboratory of Microstructures (Nanjing), Key Laboratory of Advanced Photonic and Electronic Materials of Jiangsu Province, School of Electronic Science and Engineering, Nanjing University, Nanjing, 210093, Jiangsu Province, China; 2 Department of Electronic Engineering, The Chinese University of Hongkong, Shatin, New Territories, Hong Kong, China

**Keywords:** conducting polymers, nanowires, nanotubes, polyaniline, polypyrrole, template synthesis

## Abstract

Conducting polymer nanostructures have received increasing attention in both fundamental research and various application fields in recent decades. Compared with bulk conducting polymers, conducting polymer nanostructures are expected to display improved performance in energy storage because of the unique properties arising from their nanoscaled size: high electrical conductivity, large surface area, short path lengths for the transport of ions, and high electrochemical activity. Template methods are emerging for a sort of facile, efficient, and highly controllable synthesis of conducting polymer nanostructures. This paper reviews template synthesis routes for conducting polymer nanostructures, including soft and hard template methods, as well as its mechanisms. The application of conducting polymer mesostructures in energy storage devices, such as supercapacitors and rechargeable batteries, are discussed.

## 1. Introduction

Since the discovery of the first conducting polymer, polyacetylene in 1977, the conducting polymers research field has been established and developed in an unexpectedly accelerated rate [1–5]. Conducting polymers are unique photonic and electronic functional materials owing to their high π-conjugated length, unusual conducting mechanism, and reversible redox doping/de-doping process. Conducting polymers show various promising applications, such as in transistors [6], sensors [7–10], memories [11], actuators/artificial muscles [12–14], supercapacitors [15], and lithium ionic batteries [16]. In the past decade, conducting polymers nanostructures have become a rapidly growing field of research, because they display new properties related to their nanoscale size and have greatly improved the performance of devices [8,11,17–21]. Conducting polymer nanostructures can be synthesized by several approaches, such as well-controlled solution synthesis [22–25], soft-template methods [26], hard-template methods [27,28], and electrospinning technology [29,30].

In recent years, the low carbon economy of sustainable and renewable resources has become a great challenge due to climate change and the decreasing availability of fossil fuels. It is now essential to develop new, low-cost, and environmentally friendly energy conversion and storage systems. Advances have already been made in energy storage. These include rechargeable lithium batteries and supercapacitors [31–33]. Conducting polymers having good electrochemical activity [34], such as polyaniline, polypyrrole and polythiophene, are important electrode materials for pseudo-capacitors and rechargeable lithium batteries [16,35–37]. Compared with bulk conducting polymers, conducting polymer nanostructures are expected to display improved performance in technological applications [38], because of the unique properties arising from their nanoscale size: (i) high electrical conductivity [39,40]; (ii) large specific surface area [41]; (iii) short path lengths for the transport of ions; (iv) improved cycle life due to better accommodation of the strain caused by electrochemical reaction [42,43]; (v) mixed conductive mechanism of both electronic and ionic conductivity, which lowers the interfacial impedance between electrodes and electrolyte; (vi) light weight and large ratio of specific discharge power to weight. Material chemists are attempting to design and synthesize well-structured conducting polymer nanomaterials to realize high-performance supercapacitors and rechargeable lithium batteries. Template synthesis has offered a facile, efficient, and highly controllable route to designing and synthesizing novel conducting polymer nanostructures and composites.

This paper reviews the template synthesis routes for conducting polymer nanostructures, including the soft template, hard template, and reactive template methods and mechanisms. Some selected samples are discussed, particularly with regards to designing and synthesizing fine mesostructures of conducting polymers with high performance in energy storage devices, such as supercapacitors and rechargeable batteries.

## 2. Template Directed Growth of Conducting Polymer Nanostructures

**Scheme 1.**
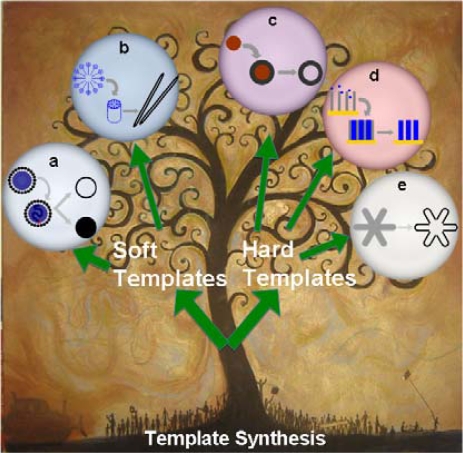
Illustration of the template synthesis of conducting polymer nanostructures: 1) soft template method and 2) hard template method. Method 1) includes **a**) microemulsion and reversed-microemulsion synthesis; and **b**) non-template (self-template) synthesis, in which monomer or oligomer forms structural micelles by themselves. Method 2) includes: **c**) physical templating against existing nanostructure of particles; **d**) structural replicate against nanochannels, the method is firstly raised by Prof. C. R. Martin; **e**) reactive template method, which clone nanostructures by the chemical reaction between template and monomers. Background of the picture is the art “tree-of-life”, by Tim Parish in 2008, which is available at: http://torrancepubliclibrary.files.wordpress.com/2009/08/tree-of-life-web.jpg.

The template synthesizing route of conducting polymer includes soft template and hard template methods. The former relies on molecular self-assembly to form nanostructures, while the latter replicates existing nanostructure by physical or chemical interactions (Scheme 1).

### 2.1. Soft Template

The Soft template synthesis, also named self-assembly method, employs micelles formed by surfactants to confine the polymerization of conducting polymers into low dimensional nanomaterials. Typical synthesis of this sort includes microemulsion polymerization and reversed-microemulsion polymerization [44] in which surfactants are involved, and the non-template (or self-template) synthesis in which the monomer or its salt forms micelles by itself.

Microemulsion (oil-in-water) polymerization produces conducting polymer nanoparticles with good control over the size of nanoparticles. The structure and concentration of surfactants and monomers are critical factors for controlling the morphological parameters of products. Jang *et al.* synthesized polypyrrole with a monodispersed size in a microemulsion with alkyl-trimethylammonium bromide cationic surfactants [45]. The size of the polypyrrole nanoparticles could be well controlled to be less than 5 nm. They found that the surfactants most suitable for microemulsion polymerization should have alkyl lengths between C6 to C16 because alkyl chains shorter than C6 have weak hydrophobic interactions, while alkyl chains longer than C16 have too high a viscosity to form self-assembled nanostructures. Monodispersed polypyrrole nanospheres were synthesized at reagent concentrations between critical the micelle concentration (CMC) I and II. Guo *et al.* used sodium dodecyl sulfate (SDS, an anionic surfactant) and HCl solution to control the morphology of polyaniline [46–48]. They found that the pH value of the solution dramatically influenced the self-assembly morphology of the products. Polyaniline in the forms of granules, nanofibers, nanosheets, rectangular submicrotubes, and fanlike/flowerlike aggregates were obtained by using different SDS and HCl concentrations.

**Figure 1.**
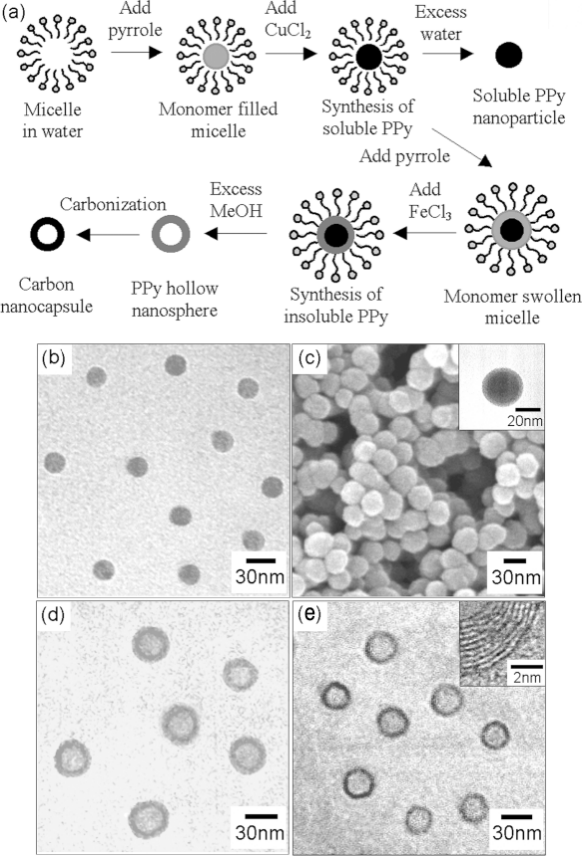
**a**) Schematic diagram of the microemulsion fabrication of Polypyrrole hollow nanospheres, and their carbon derivative**. b-e**) transmission electron microscopy (TEM) and scanning electron microscopy (SEM) images of Polypyrrole nanoparticles and hollow spheres: **b**) soluble Polypyrrole nanoparticles; **c**) linear Polypyrrole/crosslinked Polypyrrole core/shell nanoparticles; **d**) Polypyrrole nanocapsules; **e**) carbon nanocapsule derivative. Reproduced with permission from The Royal Society of Chemistry [49].

The microemulsion polymerization process can be modified to synthesize nanocapsules, nanocomposite, and mesoporous structures of conducting polymers. Jang *et al.* produced polypyrrole nanocapsules by generating a soluble polypyrrole core and a crosslinked polypyrrole shell by sequentially using initiators of different oxidation potentials [49], as shown as Figure 1. A linear polypyrrole core soluble in alcohol was produced in the first stage using copper (II) chloride with a lower oxidation potential (*E*° =+0.16 V), while an insoluble crosslinked polypyrrole shell was created in the later stage using iron (III) chloride with higher oxidation potential (*E*° = +0.77 V). Polypyrrole nanocapsules were obtained when excess methyl alcohol was added, which etched the linear polypyrrole core along with the surfactants, and the crosslinked polypyrrole shell was retained. Jang *et al.* also used surfactant-mediated interfacial polymerization (SMIP) to produce poly(3,4-ethylenedioxythiophene) (PEDOT) nanocapsules and mesocellular foams [50]. In the SMIP process, the surfactant micelles were able to capture the redox initiator due to their electrostatic interactions with cations of initiator, and this allowed the initiator to react with the monomer at the micelle/water interface, which generated hollow nanostructures of conductive polymers efficiently.

**Figure 2.**
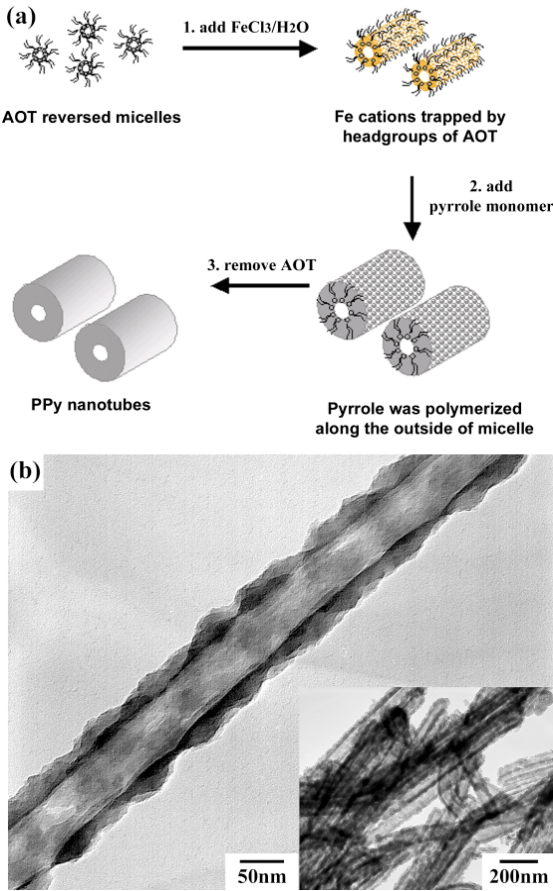
**a**) Schematic diagram of Polypyrrole nanotube fabrication using reverse microemulsion polymerization. **b**) transmission electron microscopy (TEM) image of Polypyrrole nanotubes. Reproduced with permission from The Royal Society of Chemistry [52].

Reversed microemulsion (water-in-oil) polymerization generates conducting polymer nanostructures such as monodispersed nanoparticles and nanotubes/rods, with morphology controlled by introducing the interaction between ions and surfactant. Jang *et al.* fabricated polypyrrole nanotubes through chemical oxidation polymerization in sodium bis(2-ethylhexyl) sulfosuccinate (AOT) reverse emulsions in an apolar solvent [51,52], as shown as Figure 2. AOT reverse cylindrical micelles were formed via a cooperative interaction between an aqueous FeCl_3_ solution and AOT, where FeCl_3_ aids the formation of rod-shaped micelles by decreasing the CMC II value and increasing the solvent’s ionic strength. Pyrrole monomers introduced into the reverse cylindrical micelle phase were then rapidly polymerized by iron cations along the surface of the reverse cylindrical micelles, which resulted in the formation of polypyrrole nanotubes. The residues of AOT and other reagents could be removed by thoroughly washing with excessive ethanol. In a similar method, Manohar *et al.* obtained PEDOT nanotubes [53]. Jang *et al.* obtained PEDOT nanorods by chemical oxidation polymerization of the monomer locally on the micelle surface using different reagent concentrations [54]. These researches indicated that the nanostructures of conductive polymers strongly depended on the surfactant concentration and amount of oxidizing agent in reversed microemulsion polymerization.

Surfactant gel is one kind of soft template that can guide the growth of conducting polymers. Polyaniline nanobelts were synthesized by a self-assembly process using the chemical oxidative polymerization of aniline in surfactant gel [55]. In this process, CTAB and aniline self-assembled into belt-like structures, which acted as templates for the formation of polyaniline nanobelts. The subsequent *in situ* oriented oxidative polymerization of aniline resulted in the formation of polyaniline nanobelts because of the confinement of the surfactant gel.

**Figure 3.**
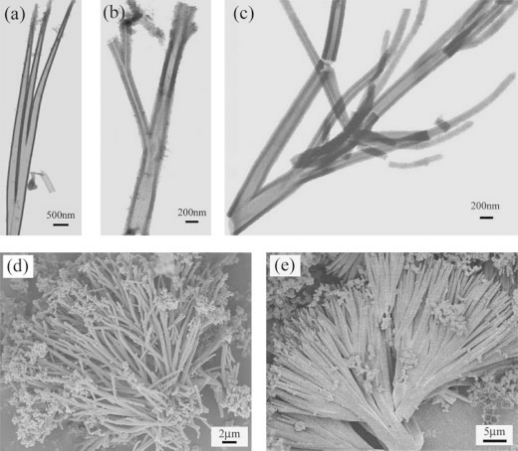
Conductive polymer nanotube junctions and their aggregated dendrites fabricated using non-templating (self-assembly) method: **a-c**) transmission electron microscopy (TEM) and **d**) scanning electron microscopy (SEM) image of Polyaniline nanotube junctions; **e**) SEM image of Polypyrrole dendrite. Reproduced with permission from Wiley-VCH Verlag [60].

Some nanostructural morphology of polyaniline could be prepared by the template-free or surfactant-free method (self-template method) [26,56]. In this synthesis, the monomer of conducting polymers or its salts form micelles by themselves, which act as templates for the formation of nanostructures. Wan *et al.* conducted a thorough research in this field regarding its universality, controllability, and self-assembly mechanism by changing the polymeric chain length, polymerization method, dopant structure, and reaction conditions. They synthesized a variety of micro/nanotubes [57–59], nanofibers, nanotube junctions (Figure 3) [60], and hollow microspheres [61] by the template-free method. The structural parameters of polyaniline nanostructures were tunable by changing dopant structure, the redox potential of the oxidant, and reaction conditions. By varying the reaction temperature or the molar ratio of dopant to aniline, the polyaniline 3D hollow spheres, nanotubes, and dendrites with nanotube junctions could be selectively produced. Some other groups also conducted relative research also [62], Guo *et al.* developed an efficient method to synthesize poly(o-toluidine) hollow spheres with controllable size and a hole in each single sphere [63,64]. The investigation of these groups shows that the template-free method, which is essentially a kind of a soft-template and self-assembly process, can be a simple and universal approach to synthesizing polyaniline micro/nanostructures [65].

The soft template method owns the advantages of low cost and large yield, which is suitable for the production in large quantities in one pot. Meanwhile, some routes that involve multi-phase solution, such as the microemulsion, reversed-microemulsion and self-template method, have great potential for synthesizing inorganic/conductive-polymer composite nanostructures by interfacial reactions. The shortage of the soft template method in energy storage devices rises from the discontinuous morphology of particles in electrode, which increases the electronic impedance in a certain extent.

### 2.2. Hard Template

The hard-template synthesis employs a physical template as a scaffold for the growth of conducting polymers. The hard template scaffold includes colloidal particles and some templates with a nanosized channel, such as anodized alumina oxide (AAO) and mesoporous silica/carbon templates [66,67].

**Figure 4.**
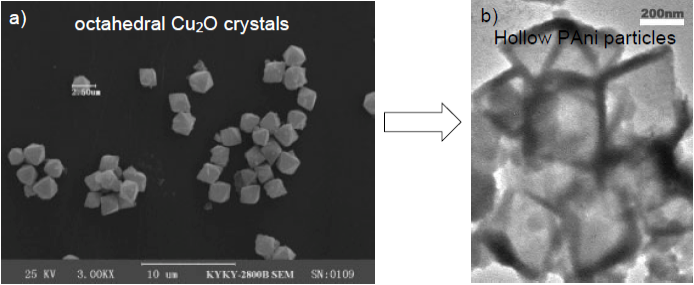
Polyaniline hollow particle fabricated by the hard template method. **a**) Scanning electron microscopy (SEM) image of Octahedral Cu_2_O crystal template; **b**) Polyaniline hollow particle replicates. Reproduced with permission from Wiley-VCH Verlag [77].

For the synthesis using micro/nanoparticles as templates, the target material is precipitated or polymerized on the surface of the template [68], which results in a core-shell structure [69,70]. After removal of the template, hollow nanocapsules or nanotubes can be obtained [71–73]. The most commonly used hard templates include monodispersed inorganic oxide nanoparticles [28,74] and polymer microspheres [75,76]. These kinds of templates are advantageous for several reasons: narrow size distribution, ready availability in relatively large amounts, availability in a wide range of sizes from commercial sources, and simplicity of synthesis using well known formulations. However, the removal of the template often affects the hollow structures. Furthermore, the post-processing for template removal is tedious. Wan *et al.* developed a template self-removing process to produce a polyaniline hollow structure with octahedral cuprous oxide as template as shown as Figure 4, which was spontaneously removed by reaction with an oxidative initiator, ammonium peroxydisulfate [77]. The method simplified the process to produce polyaniline hollow structures in a quantitative way. The only potential drawback of the method is that a reduced emeraldine form of polyaniline was produced because of the reducibility of the cuprous oxide template.

**Figure 5.**
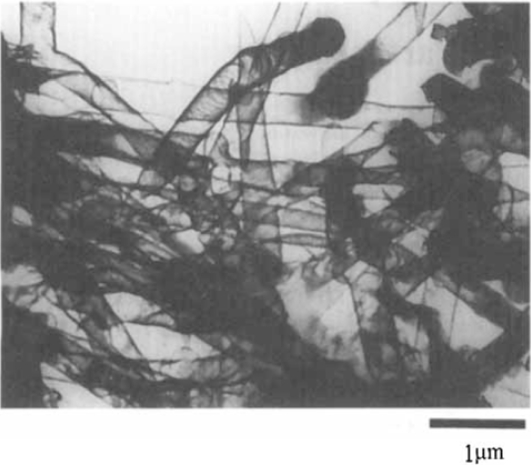
Transmission electron microscopy (TEM) image of the conductive polymer nanotubes fabricated using the AAO template. Reproduced with permission from The International Society of Electrochemistry [80].

A template with a nanosized channel can be used to produce conducting polymer nanowires/tubes with a restricted deposition/growth effect [41,78–85]. This kind of templating method was first developed by Martin [66,80], and soon became a classic method with highly controllability to produce nanowire/tube nanostructures, and most importantly its arrays. In this approach, the conducting polymer nanostructures can be formed by filling the templates through physical or electrochemical deposition [82,83,86–88]. The commonly used and commercially available templates of this sort are anodized alumina oxide membrane [81,89], radiation track-etched polycarbonate (PC) membranes [90–93], zeolite [39], and mesoporous carbon. The AAO template can be used to fabricate conducting polymer composite with well-tuned nanostructures by controlled the electrochemical deposition [94–96]. The first reported transmission electron microscopy (TEM) image of conductive polymer nanotubes using the AAO template by Martin group is shown in Figure 5. One of the most attractive advantages of this route is that the ordered array of conducting polymers nanotubes can be produced using the AAO template [97,98]. Whitesides *et al.* fabricated core-shell and segmented polymer-metal composite nanostructures by sequentially depositing polyaniline and Au via an electrochemically route [99]. Some mesoporous materials with open nanochannels can be used as template to produce conducting polymer nanofiber or its composites. Bein *et al.* prepared conducting filaments of polyaniline in the 3 nm wide hexagonal channel of the aluminosilicate MCM-41 [39]. Aniline vapor was adsorbed onto the dehydrated host. This was followed by a reaction with peroxydisulfate, leading to encapsulated polyaniline filaments. They measured the conductivity of the polymer filaments by contactless microwave absorption at 2.6 GHz. The materials showed good low-field conductivity, which demonstrated for the first time that conjugated polymers can be encapsulated in nanometer channels and still support mobile charge carriers.

### 2.3. Reactive Template and Mechanism Study

One of the hard templates with highest potential for conducting polymers synthesis is the oxidative inorganic/organic nanostructures, such as the V_2_O_5_ or MnO_2_ nanowires/fibers. This kind of template can initiate the polymerization of monomers by oxidative reactions, and then effectively transfer their morphology to the conducting polymer. By simply changing the morphology of the reactive template, different sizes and shapes of conducting polymers are possible. The reactive template method is a simple, one-step procedure, since most of the reactive templates could be converted to soluble ions in a redox reaction. As a result, no special purification steps are required to obtain the pure polymer.

Lu *et al.* obtained polypyrrole nanotubes by using a fibrillar complex of FeCl_3_ and methyl orange (MO) as the template [100]. The complex of FeCl_3_ and MO could initiate the polymerization of pyrrole monomer and direct the growth of polypyrrole into nanotubes, which self-degraded after the reaction and left azo-functionalized polypyrrole nanotubes in high yield.

**Figure 6.**
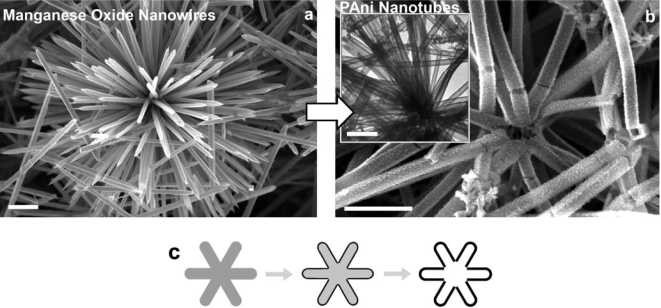
Scanning electron microscopy (SEM) images of **a**) cryptomelane-phase manganese oxide template, and **b**) resultant polyaniline nanotubes. The inset of (b) is a transmission electron microscopy (TEM) image of the polyaniline nanotubes. **c**) Schematic illustration of the formation mechanism of the polyaniline nanotubes. The scale bar is 1 μm. Reproduced with permission from Wiley-VCH Verlag [27].

Pan *et al.* developed a reactive template strategy by using manganese oxide nanowires to produce polyaniline nanotubes as shown as Figure 6 [27]. In this case, the MnO_2_ nanowires served as both oxidative polymerization initiator and physical template. The oxidation potential of MnO_2_ was sufficient to initiate the polymerization of aniline, and polyaniline films formed on the surface of the MnO_2_ nanowires as the polymerization proceeded. The morphology of the MnO_2_ nanowires was thus cloned by polyaniline, which resulted in polyaniline nanotubes with an external size and shape similar in dimensions to that of the MnO_2_ nanowire template. The reactive template strategy was simple and direct because the reactive MnO_2_ template could be converted to soluble Mn^2+^ ions during the polymerization process. As a result, no special purification steps were required to obtain the pure polymer.

**Figure 7.**
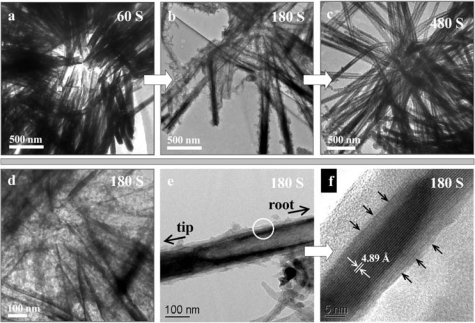
Transmission electron microscopy (TEM) images show the structural evolution during the conversion from cryptomelane phase manganese nanowires to polyaniline nanotubes after: **a**) 60 s; **b**) 180 s; and **c**) 480 s. **d**) the magnified image of the root region of (b). **e**), **f**) HRTEM (high-resolution TEM) images indicate the formation of polyaniline (shell)/manganese oxide (core) composite tube in the corrosive etching of manganese oxide. The scale bar is 1 μm. Reproduced with permission from Wiley-VCH Verlag [27].

**Figure 8.**
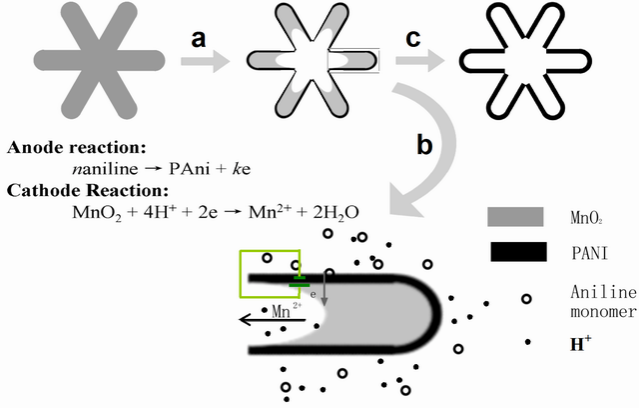
Schematic illustration of the proposed microzone galvanic cell reaction that occurs during the conversion from manganese oxide wire to polyaniline nanotube. **a**) The aniline was polymerized on a manganese oxide nanowire surface. **b**) The hollow structure developed mainly through the micro-zone galvanic-cell reaction mode. **c**) The homogeneous polyaniline tube was finally formed because the whole surface of the polyaniline (Polyaniline) thin film was almost equipotential in the micro-zone galvanic-cell reaction. Reproduced with permission from Wiley-VCH Verlag [27].

A microzone galvanic cell reaction mechanism contributes to a high quality replica in the reactive template synthesis (Figures 7 and 8, also see the Supporting Information in [27]). TEM investigation of the morphology evolution revealed that the voids of MnO_2_ were developed inside the nanowires of MnO_2_ (Figure 7). No polyaniline was polymerized in the inner surface of the voids, while polyaniline is homogeneously grown on the outer surface of MnO_2_ nanowires (with no dependence on the local MnO_2_ consumed). The reaction is schematically illustrated as the two half-cell reactions in Figure 8, which is the typical reaction mode of a microzone galvanic cell. The reaction is caused by differences in local chemical environment. This microzone galvanic cell reaction mechanism enables the high-fidelity replication of the structure of manganese by polyaniline. Furthermore, the microzone galvanic cell reaction mechanism has a great potential for the fabrication of nanostructures or novel nanocomposites.

The reactive template method presents a strong potential for shape controlling. Different polyaniline nanosizes and shapes are possible by simply changing the morphology of the MnO_2_ template, for example, Li *et al.* prepared spherical and cubic hollow structures of polyaniline and polypyrrole by using a structured MnO_2_ template [101].

In recent years, the hard-template synthesis has received increasing interest for energy storage devices, due to the following reasons: (i) hard-template synthesis has provided a strong tool to produce arrayed conductive polymer nanowires/tubes, which improve ionic change of electrode materials and electronic transport to the collector; (ii) hard-template synthesis is an easy way to produce inorganic/organic composite nanostructures, which show high performance in supercapacitors and batteries.

In general, template synthesis has offered chemists a more flexible and efficient route to synthesize well-defined conducting polymer nanostructures. Meanwhile, the nanostructure synthesized by template synthesis shows some improved physical properties that show great potential for its application in energy storage devices, for example, conductivity at room temperature. Martin and his group found that template-synthesized conducting polymer tubes or fibrils have enhanced conductivity as compared with bulk materials. The nanotubes have enhanced conductivity because the conductive polymer as synthesized has high molecular and supermolecular order. The Martin group proposed several mechanisms for the formation of the high ordering of the conductive polymer: 1) the chain of conductive polymer deposited in the nanochannels oriented according to the ordering of the polycarbonates molecules in membrane wall; 2) the polycationic conductive polymer preferentially polymerizes on the anionic sites on the pores of polycarbonates membrane; 3) the chain ordering of conductive polymer is induced by the confined synthesis into nanopores acting as nanoreactor [102–105]. Meanwhile, it was found that the conductivity is enhanced with decreasing pore diameter [17,106–111]. A dramatic change in conduction behavior from an insulating regime to a metallic regime through the critical regime as diameter decreases.

### 3. High-Power Energy Storage Devices: Supercapacitors and Batteries

**Figure 9.**
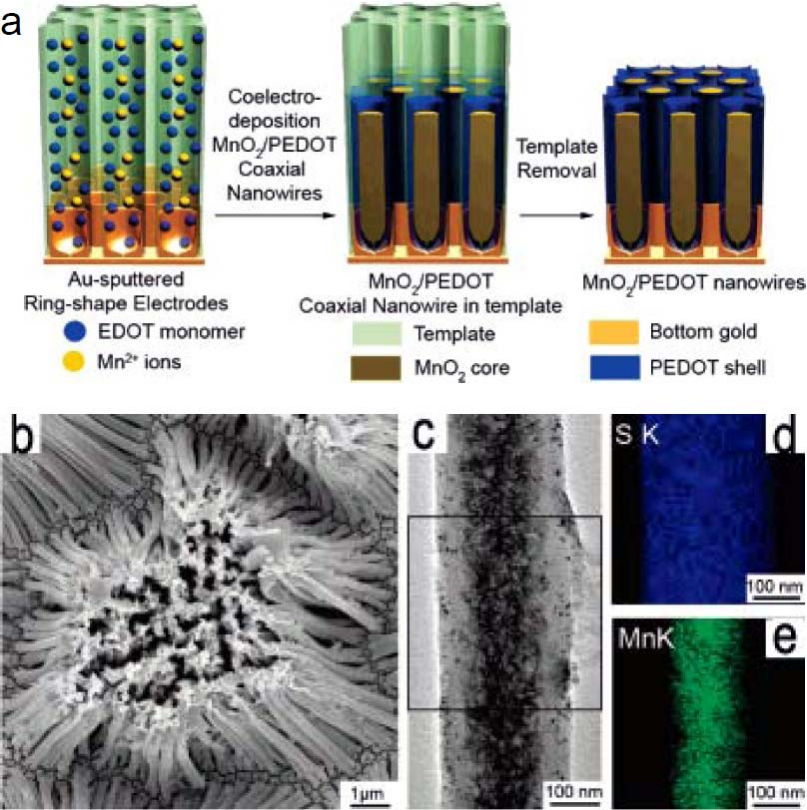
**a**) Schematic illustration of the formation mechanism of MnO_2_/PEDOT composite nanowires; **b**) Scanning electron microscopy (SEM) image of MnO_2_/PEDOT coaxial nanowires (0.75 V). **c**) Transmission electron microscopy (TEM) image from a single coaxial nanowire (0.75 V). **d** and **e**) Energy Dispersive Spectroscopy (EDS) maps of S and Mn from the boxed area in Figure 9c. Reproduced with permission from The American Chemical Society [115].

Conducting polymers are important electrode materials for electrochemical energy storage devices[112], such as supercapacitors [31] and lithium secondary batteries [113]. There are several potential advantages associated with the development of conducting polymers nanoelectrodes for these devices: (i) higher electrode/electrolyte contact area leading to higher charge/discharge rates; (ii) short path lengths for electronic transport (permitting operation with low electronic conductivity or at higher power); (iii) short path lengths for the transport of ions; and (iv) better accommodation of the strain of the electrochemical reaction to improve cycle life. Template synthesis has offered chemists a more flexible and efficient route to synthesizing well-designed conducting polymer nanostructures with improved electrochemical energy storage [114]. Some typical examples are discussed below:

Lee *et al.* synthesized the composites of PEDOT and MnO_2_ nanowires by a one step electrochemical co-deposition in an AAO template, as shown as Figure 9 [115]. The composite nanowire had a coaxial structure with PEDOT as the shell and MnO_2_ as the core. The coaxial nanowires could be used as excellent supercapacitor materials, which not only exhibited high specific capacitance values but also showed a greatly improved ability to maintain capacitance at high current density, preserving 85% of its specific capacitance as the current density increased from 5 to 25 mA/cm^2^. The well-maintained specific capacitance was mainly attributed to the short paths of ion diffusion in the nanowires, wherein the porous nature of the PEDOT shell allowed for fast ion diffusion into the MnO_2_ core. On the other hand, the highly electrical conducting PEDOT shell facilitated electron transport to the MnO_2_ core, which increased the conductivity of the coaxial nanowire. The electrochemical capacitance of nanowire materials could be fully utilized, especially for the performance at high current density, due to its well-tuned microstructure, which is crucial for high power demand when operating at high charge and discharge rates.

**Figure 10.**
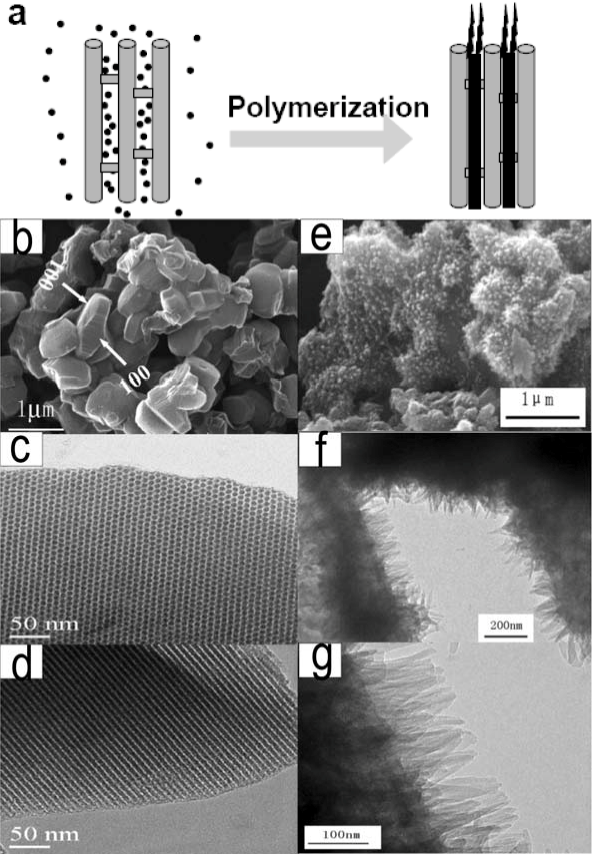
**a**) The illustration of the synthetic route of Wang to well structured conducting polymers and mesoporous carbon. **b**) SEM image of mesoporous carbon product; **c**, **d**) TEM images of mesoporous carbon seen from the [001] and [100] directions; **e**) SEM image of POLYANILINE/mesoporous carbon product; **f**, **g**) TEM images of POLYANILINE/mesoporous carbon at different magnifications. Reproduced with permission from Wiley-VCH Verlag [15].

Composites of conducting polymers and mesoporous carbon have great potential applications for energy storage devices because mesoporous carbon backbone can provide a material with good stability and increased electronic conductivity, while conducting polymers provide electrochemical activity. Moreover, the discovery that ion desolvation occurs in pores smaller than the solvated ions has led to higher capacitance for electrochemical double layer capacitors using carbon electrodes with subnanometer pores, and opened the door to designing high-energy density devices with mesoporous carbon materials. Xia *et al.* reported the growth of ordered whisker-like polyaniline on the surface of a mesoporous carbon template and its excellent supercapacitor properties [15]. The nanosize polyaniline thorns were polymerized on a mesoporous carbon surface, and formed “V-type” nanopores as show as Figure 10. These nanopores yielded high electrochemical capacitance because the “V-type” channels facilitated faster penetration of the electrolyte and the shorter diffusion length of ions within the electrode during the charge–discharge process. On the other hand, the high conductivity of polyaniline and mesoporous carbon greatly reduced energy loss and power loss at high charge–discharge current density. The specific capacitance of the polyaniline/mesoporous carbon composite was as high as 900 F g^−1^ at a charge–discharge current density of 0.5 A g^−1^ (or 1221 F g^−1^ for polyaniline, based on pure polyaniline in the composite). This was a significant progress on supercapacitor research because the capacitance value was higher than that of amorphous hydrated RuO_2_ (840 F g^−1^), while polyaniline is much cheaper than RuO_2_. Furthermore, the capacitance retention of this composite was higher than 85% when the charge–discharge current density increased from 0.5 Ag^−1^ to 5 Ag^−1^, indicating its potential high power performance while operating at high charge and discharge rates.

Arrayed conducting polymer nanotube/fibers were produced within an APA template by both physical and electrochemical deposition. It has led to greatly improved properties of rechargeable batteries. Chen *et al.* produced polyaniline nanofibers and nanotubes using a spray technique by wetting the APA template with a conducting polymers solution [116]. The nanofibers/tubes showed excellent electrochemical performance when used as a positive electrode material in lithium batteries. The discharge capacity value of the doped polyaniline nanotubes/nanofibers reached 75.7 mA h g^−1^, and retained 72.3 mA h g^−1^ (95.5% of the highest discharge capacity) in the 80th cycle. The discharge capacity of polyaniline nanotubes is much higher than the best practical discharge capacity of the commercially doped polyaniline powders (54.2 mA h g^−1^). Meanwhile, the specific discharge energy of the nanostructures reached 227 W h Kg^−1^, showing excellent storage of high specific energy for Li/polyaniline rechargeable cells. The average capacity deterioration of the nanostructural doped polyaniline was less than 0.05 mA h g^−1^ for one cycle, indicating their superior cycling capability. In addition, the nanotube electrode exhibited longer charge and discharge plateaus than the electrode composed of commercial powders. All these indicate the great potential application of polyaniline nanotubes synthesized by template method as high performance cathode-active materials for Li/polyaniline rechargeable batteries. Moreover, the composite of nanostructured polyaniline and V_2_O_5_ showed a potential application in lithium secondary battery. Li *et al.* fabricated uniform one-dimensional V_2_O_5_/polyaniline core-shell nanobelts by using V_2_O_5_ nanobelt as a reactive template [117]. The formation of the V_2_O_5_/polyaniline core-shell nanobelts was related to the *in situ* polymerization of aniline monomer through etching V_2_O_5_ nanobelts. They studied the electrochemical lithium intercalation/deintercalation of V_2_O_5_/polyaniline core-shell nanobelts and showed that the material can be used in lithium secondary batteries.

## 4. Conclusions

Climate change and the rapidly decreasing availability of fossil fuels require society to move in an accelerating speed towards the use of sustainable and renewable resources. Supercapacitor and lithium batteries are two important devices for energy storage and release. The design and bulky fabrication of fine nanostructures of conducting polymers and composites is the key to success in designing tomorrow’s high-energy and high-power devices. This review strongly suggests the use of the template method as a simple, universal, and controlled approach to fabricate novel conducting polymer nanostructures and composites. Furthermore, some cases on designing and synthesizing fine mesostructures of conducting polymer with high performance in energy storage devices, were discussed.
